# Communities, universal health coverage and primary health care

**DOI:** 10.2471/BLT.20.252445

**Published:** 2020-08-27

**Authors:** Emma Sacks, Meike Schleiff, Miriam Were, Ahmed Mushtaque Chowdhury, Henry B Perry

**Affiliations:** a Department of International Health, Johns Hopkins Bloomberg School of Public Health, 615 N. Wolfe St, E8011, Baltimore, Maryland, 21205, United States of America.; b UZIMA Foundation, Nairobi, Kenya.; c BRAC, Dhaka, Bangladesh.

## Abstract

Universal health coverage (UHC) depends on a strong primary health-care system. To be successful, primary health care must be expanded at community and household levels as much of the world’s population still lacks access to health facilities for basic services. Abundant evidence shows that community-based interventions are effective for improving health-care utilization and outcomes when integrated with facility-based services. Community involvement is the cornerstone of local, equitable and integrated primary health care. Policies and actions to improve primary health care must regard community members as more than passive recipients of health care. Instead, they should be leaders with a substantive role in planning, decision-making, implementation and evaluation. Advancing the science of primary health care requires improved conceptual and analytical frameworks and research questions. Metrics used for evaluating primary health care and UHC largely focus on clinical health outcomes and the inputs and activities for achieving them. Little attention is paid to indicators of equitable coverage or measures of overall well-being, ownership, control or priority-setting, or to the extent to which communities have agency. In the future, communities must become more involved in evaluating the success of efforts to expand primary health care. Much of primary health care has taken place, and will continue to take place, outside health facilities. Involving community members in decisions about health priorities and in community-based service delivery is key to improving systems that promote access to care. Neither UHC nor the Health for All movement will be achieved without the substantial contribution of communities.

## Résumé

La couverture maladie universelle (CMU) repose sur un solide système de soins de santé primaires. Pour rendre les soins de santé primaires efficaces, il faut les étendre aux communautés et aux ménages car la majorité de la population mondiale n'a toujours pas accès aux structures médicales offrant des services de base. Nombreux sont les éléments qui prouvent que les interventions communautaires contribuent à améliorer l'utilisation des soins de santé et les résultats cliniques lorsqu'elles font partie intégrante des services proposés au sein des établissements. L'implication des communautés constitue la clé de voûte d'un système de soins de santé primaires local, équitable et intégré. Les politiques et actions visant à le renforcer doivent tenir compte des membres des communautés, et ne pas se limiter à les considérer comme des bénéficiaires passifs de soins de santé. Au contraire, leurs dirigeants devraient jouer un rôle prépondérant dans la planification, la prise de décisions, la mise en œuvre et l'évaluation. Faire progresser la science des soins de santé primaires requiert une optimisation des cadres analytiques et conceptuels, ainsi que des questions de recherche. Les paramètres employés pour évaluer les soins de santé primaires et la CMU se concentrent souvent sur les résultats cliniques, sur les activités et moyens utilisés pour les atteindre. Peu d'attention est accordée aux indicateurs d'une couverture équitable, ou aux mesures de bien-être général, de possession, de contrôle ou de définition des priorités, ou encore à l'étendue du pouvoir d'action des communautés. À l'avenir, les communautés doivent s'engager davantage dans l'évaluation de la réussite des efforts déployés pour développer les soins de santé primaires. La plupart de ces soins ont toujours été et continueront à être prodigués en dehors des structures médicales. Impliquer les membres des communautés dans les décisions destinées à définir les priorités sanitaires et la fourniture de services communautaires est essentiel pour améliorer des systèmes qui permettront de promouvoir l'accès aux soins. Ni la CMU, ni le mouvement «Santé pour tous» ne parviendront à leurs fins sans la contribution majeure des communautés.

## Resumen

La cobertura sanitaria universal (CSU) depende de un sistema de atención primaria de salud sólido. Sin embargo, la atención primaria de salud se debe ampliar a nivel de la comunidad y de los hogares para que logre resultados efectivos, ya que gran parte de la población mundial sigue sin tener acceso a los centros de salud para recibir los servicios básicos. Existen muchas pruebas que demuestran que las intervenciones basadas en la comunidad son efectivas para mejorar el uso y los resultados de la atención de la salud cuando se integran con los servicios que se prestan en los centros de salud. La participación de la comunidad es el elemento fundamental de la atención primaria de salud local, equitativa e integrada. Las políticas y las medidas para mejorar la atención primaria de salud deben tener en cuenta que los miembros de la comunidad son más que receptores pasivos de la atención de salud. Por el contrario, deben ser líderes con una función importante en la planificación, la toma de decisiones, la implementación y la evaluación. El progreso de la ciencia en la atención primaria de salud requiere mejorar los marcos conceptuales y analíticos y los temas de investigación. Los parámetros que se usan para evaluar la atención primaria de salud y la CSU se centran en gran medida en los resultados clínicos de la salud y en los recursos y las actividades que permiten alcanzarlos. Se presta poca atención a los indicadores de cobertura equitativa o a las medidas de bienestar general, propiedad, control o establecimiento de prioridades, o a la medida en que las comunidades participan activamente. Por consiguiente, las comunidades deben participar más en la evaluación del éxito de los esfuerzos por ampliar la atención primaria de salud en el futuro. Gran parte de la atención primaria de salud siempre ha tenido y seguirá teniendo lugar fuera de los centros de salud. La participación de los miembros de la comunidad en las decisiones sobre las prioridades sanitarias y en la prestación de servicios comunitarios es fundamental para mejorar los sistemas que promueven el acceso a la atención, ya que ni la CSU ni el movimiento Salud para Todos se lograrán si las comunidades no contribuyen de manera sustancial.

## ملخص

تعتمد التغطية الصحية الشاملة (UHC) على نظام قوي للرعاية الصحية الأولية. لتحقيق النجاح، يجب توسيع نطاق الرعاية الصحية الأولية في المجتمع وعلى المستويات الأسرية نظرًا لأن الكثير من سكان العالم لا يزالوا يفتقرون إلى إمكانية الاستفادة من المرافق الصحية للحصول على الخدمات الأساسية. تشير وفرة من الأدلة أن التدخلات المجتمعية تتميز بالفعالة في تحسين الاستفادة من الرعاية الصحية، ونتائجها عندما تتكامل مع الخدمات المعتمدة على المرافق. تعد المشاركة المجتمعية بمثابة حجر الزاوية للرعاية الصحية الأولية المحلية العادلة المتكاملة. إن السياسات والإجراءات المتبعة لتحسين الرعاية الصحية الأولية يجب أن تضع في اعتبارها أفراد المجتمع على أنهم أكثر من مجرد متلقين سلبيين للرعاية الصحية. وبدلاً من ذلك، يجب أن يكونوا قادة لهم دور جوهري في التخطيط، واتخاذ القرار، والتنفيذ، والتقييم. إن الارتقاء بعلم الرعاية الصحية الأولية يتطلب إطارات مفاهيمية وتحليلية وأسئلة بحثية محسنة. تركز المقاييس المستخدمة لتقييم الرعاية الصحية الأولية والتغطية الصحية الشاملة (UHC) بشكل كبير على النتائج الصحية السريرية، والمدخلات والأنشطة المتاحة لتحقيقها. لا تستحوذ مؤشرات التغطية العادلة، أو مقاييس الرفاهية العامة، أو الملكية، أو السيطرة، أو تحديد الأولويات، أو المدى الذي تتمتع به المجتمعات المحلية بالتمثيل، إلا بالقليل من الاهتمام. في المستقبل، يجب أن تصبح المجتمعات أكثر مشاركة في تقييم نجاح الجهود المبذولة لتوسيع نطاق الرعاية الصحية الأولية. كان ولا يزال قدر كبير من الرعاية الصحية الأولية يتم دائمًا خارج المرافق الصحية. إن إشراك أفراد المجتمع في القرارات المتعلقة بالأولويات الصحية، وفي تقديم الخدمات المجتمعية، أمر أساسي لتحسين الأنظمة التي تعزز الحصول على الرعاية. لن تتحقق التغطية الصحية الشاملة (UHC)، ولا حركة "الصحة للجميع" دون مساهمة كبيرة من المجتمعات.

## 摘要

全民健康覆盖 (UHC) 取决于强大的初级卫生保健体系。世界上还有很多人无法享受卫生机构提供的基本服务,因此必须在社区和家庭大力推广初级卫生保健,才能实现全民健康覆盖。大量证据表明,社区干预结合机构服务可有效提高卫生保健服务利用率,并改善其成效。社区参与是实现地方公平和一体化初级卫生保健的基石。就初级卫生保健的改善政策和措施而言,不可将社区成员仅视为被动接受卫生保健服务的人。相反,他们应该是在规划、决策、实施和评估中发挥实质性作用的带头人。要推进初级卫生保健科学,就需要改进概念、分析框架以及研究问题。初级卫生保健和 UHC 的评估指标,主要侧重于临床卫生成效以及达成该成效的投入和举措。而很少关注公平覆盖的指标,或总体福祉、所有权、控制权或明确优先事项的措施,或社区具有的代理权程度。今后,社区必须更积极参与评估推广初级卫生保健的成功程度。许多初级卫生保健一直并将继续在卫生机构以外进行。让社区成员参与卫生工作重点决策以及社区服务,是改善体系的关键,可以让更多人获得保健机会。如果没有社区的大力协助,UHC 和全民健康都将无法实现。

## Резюме

Всеобщий охват услугами здравоохранения (ВОУЗ) зависит от наличия сильной системы первичной медико-санитарной помощи. Для достижения успеха первичную медико-санитарную помощь следует расширить на уровне сообществ и домашних хозяйств, поскольку большая часть населения мира по-прежнему не имеет доступа к учреждениям здравоохранения для получения основных услуг. Многочисленные доказательства демонстрируют, что меры, предпринимаемые на уровне сообществ, эффективны для улучшения использования услуг здравоохранения и результатов, если они объединены с услугами на базе учреждений. Участие сообщества является краеугольным камнем местной, равнодоступной и объединенной первичной медико-санитарной помощи. Политика и действия по улучшению первичной медико-санитарной помощи должны рассматривать членов сообщества как нечто большее, чем просто пассивных получателей медицинской помощи. Наоборот, сообщества должны быть лидерами, играющими существенную роль в планировании, принятии решений, реализации и оценке. Развитие научных знаний о первичной медико-санитарной помощи требует улучшенных концептуальных и аналитических механизмов и исследовательских вопросов. Показатели, используемые для оценки первичной медико-санитарной помощи и всеобщего охвата услугами здравоохранения, в основном сосредоточены на клинических результатах в отношении здоровья, а также на затрачиваемых на это ресурсах и мероприятиях по их достижению. Мало внимания уделяется индикаторам справедливого охвата, измерению общего благосостояния, сопричастности, контроля или определения приоритетов или тому, насколько у сообщества имеется возможность действовать. В будущем сообщества необходимо более активно привлекать к оценке успеха усилий по расширению первичной медико-санитарной помощи. Большая часть первичной медико-санитарной помощи всегда оказывалась и будет оказываться вне учреждений здравоохранения. Вовлечение членов сообществ в решения о приоритетах сферы здравоохранения и о предоставлении услуг на уровне сообщества является ключевым фактором в усовершенствовании систем, способствующих улучшению доступа к медицинской помощи. Ни всеобщего охвата услугами здравоохранения, ни движения «Здоровье для всех» невозможно достичь без существенного вклада сообществ.

## Introduction

The achievement of universal health coverage (UHC) depends on a strong primary health-care system that can provide essential health services for the entire population.^1^ Primary health care is especially important for people who have limited access to high-quality health care because they are socially or geographically disadvantaged.^2^ Although primary health care offers a feasible and equitable route to UHC,^3^ success depends on expanding primary health care at community and household levels because, for much of the world’s population, local health facilities are still too far away to ensure convenient access to basic health services. In a 2017 report on UHC, the World Health Organization (WHO) and the World Bank concluded that over half of the world’s population lacks access to basic health services and that over 100 million people are forced into poverty annually because of health expenses, including the cost of transportation.^2^ Although improving the quality of facility-based care is necessary and may itself lead to some increase in utilization,^4^ the reality is that the cost (in terms of time, effort and money) of reaching distant facilities means that, for the foreseeable future, UHC cannot be achieved through health facilities alone. There is abundant evidence that community-based services are effective in improving health care utilization and outcomes, especially for maternal, newborn and child health, when they are integrated with facility-based services.^5^ Furthermore, primary health care needs to be comprehensive, of a high quality, people-centred, affordable and truly accessible to all.^6^

The Declaration of Alma-Ata asserts that primary health care can meet most of an individual’s health needs through the basic preventive, promotive, curative and rehabilitative care provided by low-level health workers (including community health workers, CHWs).^7^ These workers can function in teams close to people’s homes and often outside of health facilities. In a well-coordinated primary health-care system that truly operates across all health-care levels, patients with conditions that require specialist care can be referred to a higher care level, as needed.^7^ The Declaration advocates “community self-reliance and participation to organize, plan, operationalize and control health services and address the social determinants of health.”^7^ In 2018, in acknowledgement of the continued lack of universal primary health care, the Alma-Ata principles were reaffirmed at a global conference in Astana, Kazakhstan.^8^

Communities are “groups of families, individuals and other types of networks and social circles that provide support and are often the unit on which health activities are organized and focused.”^9^ Data show that community members and community-based organizations can be effective at identifying health priorities, addressing health concerns, managing financial and personal processes at the local level, and evaluating health systems and holding them to account. Local organizations trusted by the community can also be essential for guiding interventions aimed at behavioural modification and for facilitating adaptation to changing environmental, demographic and epidemiological conditions.^10^

Communities can consist of a wide and diverse set of actors, from geographically defined groups and local governance structures to users of health services. Communities are difficult to study, they are not monolithic or homogeneous and they can even be oppressive when conformity is demanded or local elites are in control. Nevertheless, they are entities with agency that must be engaged with by the formal health system.^11^ Acknowledging this lack of homogeneity entails recognizing the unique nature of participating community members and organizations, each with their own capabilities, resources, needs and interests.^12^

Some communities are transient, mobile or even virtual.^10^ They may change over time, with shifting membership, scope and priorities. In addition, changes may occur within community organizations as skills are gained and new challenges are tackled. Thus, new research approaches may be required to assess the attributable impact of community involvement in primary health care and the changes it can bring about.^13^ Communities may also have longstanding traditional power structures that do not promote the inclusion of marginalized people, such as women, ethnic and linguistic minorities, oppressed tribes or castes and the most economically disadvantaged. Addressing inequity requires paying explicit attention to power, for example by recognizing previously unacknowledged barriers to community participation and seeking types of knowledge that have not historically been given primacy.^14^^,^^15^

Individuals and communities – the most important stakeholders in the health system – must be enabled to demand a health-care system that is responsive to their needs and concerns and that works collaboratively to improve their health and well-being.^16^ Consequently, policies and actions for improving primary health care must regard community members as more than passive recipients of health care. Instead, they should be seen as leaders who have a substantive role from the beginning of planning to decision-making, implementation, evaluation and evidence-based iterative learning. This includes addressing power imbalances that limit the ability of communities to participate and restrict which community members can participate, while acknowledging differences in power and civic participation within communities.^17^ Powerful community members may monopolize the process of community engagement for their own gain, thereby reinforcing existing intracommunity power asymmetries. Addressing internal and external power imbalances is difficult; a conscious effort must be made to promote equitable involvement by the whole community. Moreover, some community members may be preoccupied with other concerns and have little time or resources to spare. Nevertheless, identifying the priorities of different community members, including those not typically consulted, through rigorous participatory research not only increases enthusiasm in the community but also respects local knowledge of urgent needs and capabilities.^18^

## Community involvement 

Community involvement is the cornerstone for developing local, equitable and integrated primary health care.^9^ The pillars of primary health care include: (i) empowered people and communities; (ii) a focus on equity; and (iii) multisectoral policy and action.^6^^,^^7^ Successful primary health care requires evidence-based interventions at the community level (by outreach teams from health facilities as well as trained community members) coupled with deliberate efforts to provide care for marginalized people. Community members and organizations can have active roles in providing services, promoting healthy behaviour and linking people to care (Table 1).

**Table 1:** Selected programmes promoting community involvement in primary health care, worldwide, 1994–present

Characteristic	Programme			
	CORE Group Polio Project^19^	Ethiopia’s health extension programme^20^	Sierra Leone’s participatory community-based health information system^21^	Local health administration by communities (CLAS)^a^^22^
Timeframe	1999 to present	2003 to present	2015 to present	1994 to 2008^a^
Context	Rural and Muslim communities in Uttar Pradesh, India	Rural communities in Ethiopia	Slums in Freetown, Sierra Leone	Nationally in Peru
Challenge	Low vaccination rates associated with communities’ lack of trust in a polio eradication campaign and in the government health system	Lack of healthy behaviour change by households despite the deployment of a national cadre of professional CHWs	Routine health records and information incomplete and underutilized	Health priorities and resource allocation had been established without local input
Main actors	CORE Group Polio Project (a consortium of NGOs with national technical input) and community leaders	Government of Ethiopia and a large volunteer women’s development army	Government of Sierra Leone, NGOs and community development groups	Government of Peru and legal local entities created to oversee health budgets and activity (i.e. CLASs)
Community’s role	Sharing community concerns and collaborating with community leaders to identify solutions	Volunteers work with their neighbours to teach and provide a role model for basic health and sanitation behaviours	CHWs collect health information, which is reviewed by community data review committees at bimonthly meetings	Community control over budgeting and the distribution of funds
Outcome	Increased participation in and understanding of polio eradication activities, expanded health services and greater government responsiveness to community health needs	“Model household” status achieved by many throughout the country	Increased community capacity to use data and take the appropriate actions	Transparent financial management and decentralized priority-setting

CHW: community health worker; CLAS: *comunidades locales de administración en salud*; NGO: nongovernmental organization.

^a^ The programme was modified from its original form in 2008.

One example of the stalemate that health interventions can encounter when they lack community support and trust was a polio eradication initiative in Uttar Pradesh, India, where pockets of persistently unvaccinated children impeded progress towards elimination of the disease. Intensive community engagement by the CORE Group Polio Project,^19^ which started in 1999, ultimately led to a broader range of maternal and child health services being provided for marginalized and predominantly Muslim families who had not participated in previous polio immunization campaigns. In effect, collaboration with the community and the actions taken in response to their specific needs resulted in greater participation.^19^

Addressing the burden of maternal and neonatal conditions, infectious and chronic, noncommunicable diseases and the drivers of these disorders, such as pollution, migration, conflict and climate change, requires action at global, national, subnational and local levels.^9^ However, change must begin in households. In Ethiopia, a health extension programme trained and deployed 38 000 paid CHWs between 2003 and 2008.^20^ Soon after, the programme set up a women’s development army of 3 million volunteers who focused on community action and behavioural change to promote clean water, sanitation, good nutrition and healthy behaviours. Each volunteer worked with a group of five female neighbours to establish the conditions necessary for them to achieve “model household” status.^23^ However, even in a large, institutionalized programme like the one in Ethiopia, the needs of CHWs must be recognized and properly provided for. High levels of stress and distress were observed among volunteers, from whom, “much was asked but to whom little was given.”^23^

High-quality health systems must include mechanisms for monitoring, evaluating and adapting.^4^ In particular, ensuring good primary health-care coverage requires robust documentation on service utilization and population health, especially for marginalized populations and places affected by disease outbreaks, conflicts or other disasters.^8^ Participatory, community-based, health information systems can complement facility-based records in providing better understanding of health among often-overlooked groups.^21^ In the slums of Freetown, Sierra Leone, community data review committees used data from a participatory, community-based, health information system to recommend changes and to hold health facilities and governments to account.^21^

Although community groups may be involved in health planning or implementation in some capacity, unless they have authority and resources, they may have little power to set priorities, tailor interventions or effect change. In 1994, the government of Peru embarked on a health system reform programme that aimed to increase access to primary health care through decentralization and community participation by creating administrative entities called *Comunidades Locales de Administración en Salud* (CLASs; local health administration communities).^22^ These groups managed the local health budget, oversaw health service delivery and facilitated community development projects. Official contracts between community groups and the national government can help ensure resources are continuously available but frequent contract renewals, variations in the budget and weak oversight can threaten these partnerships.^22^ Further, the way individuals are selected for the local management committee will have implications for its representativeness and for the community’s willingness to accept group decisions.

## Metrics and frameworks

The metrics used for evaluating primary health care and UHC largely focus on clinical health outcomes and the inputs and activities necessary for achieving them. Frequently, less attention is paid to indicators of equitable coverage or other measures of overall well-being, ownership, control and priority-setting within the health-care system, or to the extent to which communities have agency.^24^ With some notable exceptions,^25^^,^^26^ efforts to engage communities in assessing and improving the quality and coverage of primary health-care programmes have not been well documented. In the future, more comprehensive metrics need to be used when evaluating the success of efforts to improve primary health care and UHC. Metrics for consideration include: (i) the degree to which communities contribute to formulating programme priorities; (ii) the degree to which community members are involved in supervising CHWs and the effect this supervision has on their performance; (iii) the presence and effectiveness of committees responsible for overseeing local health facilities; and (iv) the level of engagement of volunteers in community health activities.

Advancing the science of primary health care requires better conceptual and analytical frameworks and research questions. Many scholars, policy-makers and programme implementers have noted that existing frameworks have a limited ability to address community health questions and that new approaches to obtaining evidence are needed.^10^^,^^13^ The conceptual framework proposed by WHO’s Commission on Social Determinants of Health provides a valuable way of monitoring and understanding feedback loops in the social determinants of health and emphasizes the context in which people and interventions are operating.^27^ However, it lacks an explicit focus on the role of the community in addressing inequities. Recently, a proposed expansion of WHO’s building blocks framework differentiated community-based service delivery from community mobilization and organization. The proposed expansion also called for greater recognition of nontraditional aspects of the health system, such as social capital, intersectoral partnerships, local governance, equitable financing, community information and data systems, and of the role of households in producing and maintaining health.^9^ Guiding frameworks, such as the Primary Health Care Performance Initiative’s conceptual framework,^28^ also expanded many of WHO’s building blocks (such as the service delivery portion of the logic model) to tease out nuances in the processes necessary for achieving the desired community outputs and for improving population health. Finally, in 2018 the Declaration of Astana’s operational framework expanded the vision of the 1978 Alma-Ata Declaration to include a set of “levers” for community engagement and primary health care-oriented research.^29^

Much of primary health care has taken place, and will continue to take place, outside health facilities, often in homes (where mothers and families usually care for ill children) and at local community health posts. Involving community members in decisions about health priorities and strategies for service delivery is key to improving systems that ensure access to care.^30^ Even in challenging circumstances, such as in the urban slums of Freetown, Sierra Leone, which were dealing with Ebola virus disease and cholera epidemics, local neighbourhood committees supported CHWs and primary health-care activities.^21^ More research is needed into: (i) the role community engagement plays in fostering trust in local health services; (ii) whether local epidemiological data gathered by CHWs and given to communities can improve healthy behaviours and the utilization of health services; (iii) how best to distribute the responsibility and burden of community engagement among community members; and (iv) which policies can increase community participation. The critical roles of health education and of social, behavioural and structural interventions should not be underestimated. More investment is needed in health policy and systems research to identify the larger societal and contextual determinants of health and health systems dynamics.

## Building the evidence

Achieving UHC and Health for All, as proposed in the Alma-Ata Declaration, requires the entire population to have access to high-quality, basic and essential services and protection from financial harm.^2^ Patients who feel their needs are not being met, that they are being mistreated by the health system or that the quality of the service is not worth its cost may not seek further care and may discourage others from seeking care.^31^ As a patient’s experience of care quality depends on expectations, the views of community members and health service beneficiaries are paramount for setting the context within which the quality of a health system can be assessed and improved. Moreover, the provision of health care to everyone through primary health care entails reducing, if not entirely eliminating, health-related inequities. Access to health care must be evaluated in multiple domains, such as geographical accessibility, financial affordability, and patient and provider acceptability.^32^^,^^33^ Any examination of the effect of a programme on health equity must not only measure the equitability of service utilization in terms of the users’ economic status but must also take into account social parameters, such as ethnicity, gender and educational level.^34^ Although community-based primary health care can be more equitable than facility-based care, equitability must be monitored over time as programmes evolve, secular trends occur, and the circumstances and preferences of community groups and members change.^35^

In 2017, an expert global panel that reviewed a synthesis of the evidence on community-based primary health care recommended that it should be a priority in any primary health-care strategy.^36^ In addition, a modelling study based on evidence about the effectiveness of interventions for mothers and their children indicated that expanding coverage of evidence-based community services would save more lives than expanding coverage only of the services that must be provided at health centres and hospitals.^37^ The case for integrated, multilevel systems is further strengthened by reports that sustainable, effective, health-care programmes require collaboration between the formal health sector and communities.^38^ There is also strong evidence supporting programmes that integrate health services with organizations that promote health education and empowerment, such as women’s groups.^39^^–^^41^ Another example of the role communities can play is participation in management and oversight committees for health and district planning, where community members can make up the majority, or even the entirety, of governing boards that provide practical guidance and have decision-making authority.^42^ In the United States of America, such committees serve as the boards of directors of federally qualified health centres, of which there are now more than 6000 serving over 20 million people.^43^ In Peru, CLAS committees oversee the activities of primary health care centres and their outreach programmes in one third of the country.^44^

Improving primary health care requires a multisectoral approach that involves addressing the social, physical and structural determinants of health that the health system cannot address effectively itself, such as poverty, educational inequalities, gender inequities, access to water, sanitation and a hygienic environment, safe and reliable transport, and government policies that promote health. Consequently, the complexity of population health demands that we look beyond formal health facilities and beyond the health sector itself when seeking improvements.^45^ Programmes that take a holistic approach to health – for example, women-focused poverty alleviation programmes that include both government and civil society institutions – have produced clear improvements in child mortality and reduced inequity.^46^^,^^47^ In addition, CHWs (who are known by many names and acronyms) are a recognizable and often essential part of health systems. In many situations, they serve as the primary providers of community-based primary health care. The many elements of service delivery provided by health workers in the community must be planned, funded, regulated, monitored, evaluated and improved.^48^ Thus, as CHW programmes for improving primary health care increase in scale, more attention should be given to health workers’ competence, the sustainability of their roles and proper compensation. A recent review of national programmes for CHWs provided comprehensive details about 29 initiatives identified.^49^

Evidence linking community engagement with improvements in health outcomes still remains “situation-specific…unpredictable, and not generalizable.”^50^ Nevertheless, lessons can be learnt from research on smaller, community-focused projects: investigating how actions undertaken within primary health-care systems produce the desired activities and outputs can be instructive for designing and implementing larger-scale systems. Community meetings and local organizations are critical for reaching service delivery targets and for optimizing improvements in health, especially when they form part of a dynamic and iterative agenda that changes in response to ongoing dialogue and regularly reflects progress and revised goals.^51^

The extent to which communities are included, and play a leading role, in progress towards UHC is political. Community involvement is essential for maximizing coverage of primary health care and achieving Health for All.^52^ The most recent *Disease Control Priorities* report stated that, “Without initiatives to help community health platforms flourish around the world, the health gains promised by interventions will cost more and deliver less…. With the availability of local data, local forums for sharing data, and local multisectoral stakeholder engagement, the solutions will work better and deliver more.”^44^ To deliver UHC for all efficiently, primary health care must be advanced in partnership with communities.

## Conclusion

Community involvement in primary health care is essential for achieving UHC and Health for All because, in practice, primary health care begins in the household and community. Similarly, research to guide, and demonstrate the value of, an orientation towards communities also requires meaningful community leadership and participation at all stages in the process of increasing access to, and improving the quality of, health services. Neither UHC nor Health for All will be achieved without the substantial contribution of communities.

## References

[1] Binagwaho A, Adhanom Ghebreyesus T. Primary healthcare is cornerstone of universal health coverage. BMJ. 2019 6 3;365(June):l2391. 10.1136/bmj.l2391 31160322

[2] Tracking universal health coverage: 2017 global monitoring report. Geneva and Washington, DC: World Health Organization and The World Bank; 2017. Available from: http://documents.worldbank.org/curated/en/640121513095868125/pdf/122029-WP-REVISED-PUBLIC.pdf [cited 2020 Jan 1].

[3] The world health report 2008: primary health care now more than ever. Geneva: World Health Organization; 2008. Available from: http://www.who.int/whr/2008/en/ [cited 2020 Jan 1].

[4] Kruk ME, Gage AD, Arsenault C, Jordan K, Leslie HH, Roder-DeWan S, et al. High-quality health systems in the Sustainable Development Goals era: time for a revolution. Lancet Glob Health. 2018 11;6(11):e1196–252. 10.1016/S2214-109X(18)30386-3 30196093 PMC7734391

[5] Rosato M, Laverack G, Grabman LH, Tripathy P, Nair N, Mwansambo C, et al. Community participation: lessons for maternal, newborn, and child health. Lancet. 2008 9 13;372(9642):962–71. 10.1016/S0140-6736(08)61406-3 18790319

[6] Langlois E, Barkley S, Kelley E, Ghaffar A. Advancing the science and practice of primary health care as a foundation for universal health coverage: a call for papers. Bull World Health Organ. 2019;97(8):515–515A. 10.2471/BLT.19.239889

[7] Declaration of Alma Ata. International conference on primary health care, Alma-Ata, USSR, 6–12 September 1978. Geneva: World Health Organization; 1978. Available from: https://www.who.int/publications/almaata_declaration_en.pdf?ua=1 [cited 2020 Jan 1].

[8] Declaration of Astana. Geneva: World Health Organization; 2018. Available from: https://www.who.int/docs/default-source/primary-health/declaration/gcphc-declaration.pdf [cited 2020 Jan 1].

[9] Sacks E, Morrow M, Story WT, Shelley KD, Shanklin D, Rahimtoola M, et al. Beyond the building blocks: integrating community roles into health systems frameworks to achieve health for all. BMJ Glob Health. 2019 6 22;3 Suppl 3:e001384. 10.1136/bmjgh-2018-001384 31297243 PMC6591791

[10] Sacks E, Swanson RC, Schensul JJ, Gleave A, Shelley KD, Were MK, et al. Community involvement in health systems strengthening to improve global health outcomes: a review of guidelines and potential roles. Int Q Community Health Educ. 2017 7;37(3-4):139–49. 10.1177/0272684X17738089 29086630

[11] Shore N, Ford A, Wat E, Brayboy M, Isaacs ML, Park A, et al. Community-based review of research across diverse community contexts: key characteristics, critical issues, and future directions. Am J Public Health. 2015 7;105(7):1294–301. 10.2105/AJPH.2015.302588 25973834 PMC4463398

[12] Jewkes R, Murcott A. Community representatives: representing the “community”? Soc Sci Med. 1998 4;46(7):843–58. 10.1016/S0277-9536(97)00209-8 9541070

[13] George AS, LeFevre AE, Schleiff M, Mancuso A, Sacks E, Sarriot E. Hubris, humility and humanity: expanding evidence approaches for improving and sustaining community health programmes. BMJ Glob Health. 2018 6 15;3(3):e000811. 10.1136/bmjgh-2018-000811 29946489 PMC6014224

[14] Sriram V, Topp SM, Schaaf M, Mishra A, Flores W, Rajasulochana SR, et al. 10 best resources on power in health policy and systems in low- and middle-income countries. Health Policy Plan. 2018 5 1;33(4):611–21. 10.1093/heapol/czy008 29471544

[15] Dalglish SL, Rodríguez DC, Harouna A, Surkan PJ. Knowledge and power in policy-making for child survival in Niger. Soc Sci Med. 2017 3;177:150–7. 10.1016/j.socscimed.2017.01.056 28167340

[16] Taylor DC, Taylor CE, Taylor JO. Empowerment on an unstable planet: from seeds of human energy to a scale of global change. Oxford: Oxford Scholarship Online Press; 2015.

[17] George AS, Mehra V, Scott K, Sriram V. Community participation in health systems research: a systematic review assessing the state of research, the nature of interventions involved and the features of engagement with communities. PLoS One. 2015 10 23;10(10):e0141091. 10.1371/journal.pone.0141091 26496124 PMC4619861

[18] Ahmed SM, Hossain A, Khan MA, Mridha MK, Alam A, Choudhury N, et al. Using formative research to develop MNCH programme in urban slums in Bangladesh: experiences from MANOSHI, BRAC. BMC Public Health. 2010 11 2;10(1):663. 10.1186/1471-2458-10-663 21044335 PMC3091574

[19] Solomon R. Involvement of civil society in India’s polio eradication program: lessons learned. Am J Trop Med Hyg. 2019 10;101(4_Suppl) Suppl:15–20. 10.4269/ajtmh.18-0931 31760980 PMC6776100

[20] Banteyerga H. Ethiopia’s health extension program: improving health through community involvement. MEDICC Rev. 2011 7;13(3):46–9. 10.37757/MR2011V13.N3.11 21778960

[21] O’Connor EC, Hutain J, Christensen M, Kamara MS, Conteh A, Sarriot E, et al. Piloting a participatory, community-based health information system for strengthening community-based health services: findings of a cluster-randomized controlled trial in the slums of Freetown, Sierra Leone. J Glob Health. 2019 6;9(1):010418. 10.7189/jogh.09.010418 30842881 PMC6394878

[22] Altobelli L. Case study of CLAS in Peru: opportunity and empowerment for health equity. Franklin and Lima: Future Generations and Ministry of Health of Peru; 2008 Available from: https://www.future.edu/wp-content/uploads/2018/06/2008-08-case-study-clas-peru-report.pdf [cited 2020 Jan 1].

[23] Maes K, Closser S, Tesfaye Y, Gilbert Y, Abesha R. Volunteers in Ethiopia’s women’s development army are more deprived and distressed than their neighbors: cross-sectional survey data from rural Ethiopia. BMC Public Health. 2018 2 14;18(1):258. 10.1186/s12889-018-5159-5 29444660 PMC5813408

[24] About PHCPI. The Primary Health Care Performance Initiative [internet]. Primary Health Care Performance Initiative; 2020. Available from: https://improvingphc.org/about-phcpi [cited 2020 Jan 1].

[25] Chávez D, Claure M, Moshman H, Robinson NC, LLanque R, Perry HB. Implementing the census-based, impact-oriented approach to comprehensive primary health care over three decades in Montero, Bolivia: 1. Program description. Prev Med Commun Health. 2020 5 25;3:1–7.

[26] Chávez D, Claure M, Moshman H, Robinson NC, LLanque R, Perry HB. Implementing the census-based, impact-oriented approach to comprehensive primary health care over three decades in Montero, Bolivia: 2. Program achievements, including long-term trends on mortality of children and mothers. Prev Med Commun Health. 2020 5 25;3:1–6.

[27] Commission on Social Determinants of Health. Closing the gap in a generation: health equity through action on the social determinants of health. Geneva: World Health Organization; 2008. 10.1016/S0140-6736(08)61690-6 18994664

[28] The PHPCI conceptual framework [internet]. Primary Health Care Performance Initiative; 2020. Available at: https://improvingphc.org/phcpi-conceptual-framework [cited 2020 Jan 1].

[29] Primary health care: transforming vision into action. Operational framework. Draft for consultation. Geneva: World Health Organization; 2018. Available from: https://www.who.int/docs/default-source/primary-health-care-conference/operational-framework.pdf?sfvrsn=6e73ae2a_2 [cited 2020 Jan 1].

[30] Primary health care. Key facts [internet]. Geneva: World Health Organization; 2019. Available from: https://www.who.int/news-room/fact-sheets/detail/primary-health-care [cited 2020 Jan 1].

[31] Larson E, Sharma J, Bohren MA, Tunçalp Ö. When the patient is the expert: measuring patient experience and satisfaction with care. Bull World Health Organ. 2019 8 1;97(8):563–9. 10.2471/BLT.18.225201 31384074 PMC6653815

[32] Perry HB, King-Schultz LW, Aftab AS, Bryant JH. Health equity issues at the local level: socio-geography, access, and health outcomes in the service area of the Hôpital Albert Schweitzer-Haiti. Int J Equity Health. 2007 8 1;6(1):7. 10.1186/1475-9276-6-7 17678540 PMC1971052

[33] Leonard KL, Mliga GR, Haile Mariam D. Bypassing health centres in Tanzania: revealed preferences for quality. J Afr Econ. 2002;11(4):441–71. 10.1093/jae/11.4.441

[34] Kabia E, Mbau R, Oyando R, Oduor C, Bigogo G, Khagayi S, et al. “We are called the et cetera”: experiences of the poor with health financing reforms that target them in Kenya. Int J Equity Health. 2019 6 24;18(1):98. 10.1186/s12939-019-1006-2 31234940 PMC6591805

[35] Schleiff M, Kumapley R, Freeman PA, Gupta S, Rassekh BM, Perry HB. Comprehensive review of the evidence regarding the effectiveness of community-based primary health care in improving maternal, neonatal and child health: 5. equity effects for neonates and children. J Glob Health. 2017 6;7(1):010905. 10.7189/jogh.07.010905 28685043 PMC5491949

[36] Black RE, Taylor CE, Arole S, Bang A, Bhutta ZA, Chowdhury AMR, et al. Comprehensive review of the evidence regarding the effectiveness of community-based primary health care in improving maternal, neonatal and child health: 8. summary and recommendations of the expert panel. J Glob Health. 2017 6;7(1):010908. 10.7189/jogh.07.010908 28685046 PMC5475312

[37] Black RE, Levin C, Walker N, Chou D, Liu L, Temmerman M; DCP3 RMNCH Authors Group. Reproductive, maternal, newborn, and child health: key messages from Disease Control Priorities 3rd Edition. Lancet. 2016 12 3;388(10061):2811–24. 10.1016/S0140-6736(16)00738-8 27072119

[38] Perry HB, Rassekh BM, Gupta S, Freeman PA. Comprehensive review of the evidence regarding the effectiveness of community-based primary health care in improving maternal, neonatal and child health: 7. shared characteristics of projects with evidence of long-term mortality impact. J Glob Health. 2017 6;7(1):010907. 10.7189/jogh.07.010907 28685045 PMC5491946

[39] Manandhar DS, Osrin D, Shrestha BP, Mesko N, Morrison J, Tumbahangphe KM, et al.; Members of the MIRA Makwanpur trial team. Effect of a participatory intervention with women’s groups on birth outcomes in Nepal: cluster-randomised controlled trial. Lancet. 2004 9 11-17;364(9438):970–9. 10.1016/S0140-6736(04)17021-9 15364188

[40] Perry H, Morrow M, Borger S, Weiss J, DeCoster M, Davis T, et al. Care groups I: an innovative community-based strategy for improving maternal, neonatal, and child health in resource-constrained settings. Glob Health Sci Pract. 2015 9 15;3(3):358–69. 10.9745/GHSP-D-15-00051 26374798 PMC4570011

[41] Prost A, Colbourn T, Seward N, Azad K, Coomarasamy A, Copas A, et al. Women’s groups practising participatory learning and action to improve maternal and newborn health in low-resource settings: a systematic review and meta-analysis. Lancet. 2013 5 18;381(9879):1736–46. 10.1016/S0140-6736(13)60685-6 23683640 PMC3797417

[42] Scott K, George AS, Harvey SA, Mondal S, Patel G, Sheikh K. Negotiating power relations, gender equality, and collective agency: are village health committees transformative social spaces in northern India? Int J Equity Health. 2017 9 15;16(1):84. 10.1186/s12939-017-0580-4 28911327 PMC5599900

[43] Chang CH, Bynum JPW, Lurie JD. Geographic expansion of federally qualified health centers 2007–2014. J Rural Health. 2019 6;35(3):385–94. 10.1111/jrh.12330 30352132 PMC6478577

[44] Sherry M, Ghaffar A, Bishai D. Community platforms for public health interventions. In: Jamison D, Gelband H, Horton S, et al., editors. Disease control priorities: improving health and reducing poverty. 3rd ed. Washington, DC: The World Bank; 2018:267–83.

[45] Rasanathan K, Bennett S, Atkins V, Beschel R, Carrasquilla G, Charles J, et al. Governing multisectoral action for health in low- and middle-income countries. PLoS Med. 2017 4 25;14(4):e1002285. 10.1371/journal.pmed.1002285 28441387 PMC5404752

[46] Chowdhury AMR, Bhuiya A. Do poverty alleviation programmes reduce inequitities in health? The Bangladesh experience In: Leon D, Walt G, editors. Poverty inequality and health: an international perspective. Oxford: Oxford University Press; 2000:312–31.

[47] Bhuiya A, Chowdhury M. Beneficial effects of a woman-focused development programme on child survival: evidence from rural Bangladesh. Soc Sci Med. 2002 11;55(9):1553–60. 10.1016/S0277-9536(01)00287-8 12297241

[48] Schneider H, Lehmann U. From community health workers to community health systems: time to widen the horizon? Health Syst Reform. 2016 4 2;2(2):112–18. 10.1080/23288604.2016.1166307 31514640

[49] Perry H. Health for the people: national community health worker programs from Afghanistan to Zimbabwe. Washington, DC: United States Agency for International Development; 2020. Available from: https://pdf.usaid.gov/pdf_docs/PA00WKKN.pdf [cited 2020 May 11].

[50] Rifkin SB. Examining the links between community participation and health outcomes: a review of the literature. Health Policy Plan. 2014 9;29 Suppl 2:ii98–106. 10.1093/heapol/czu076 25274645 PMC4202913

[51] Marcil L, Afsana K, Perry HB. First steps in initiating an effective maternal, neonatal, and child health program in urban slums: the BRAC Manoshi project’s experience with community engagement, social mapping, and census taking in Bangladesh. J Urban Health. 2016 2;93(1):6–18. 10.1007/s11524-016-0026-0 26830423 PMC4794453

[52] Tangcharoensathien V, Mills A, Patcharanarumol W, Witthayapipopsakul W. Universal health coverage: time to deliver on political promises. Bull World Health Organ. 2020 2 1;98(2):78–78A. 10.2471/BLT.20.250597 32015572 PMC6986228

